# Integrative Study Supports the Role of Trehalose in Carbon Transfer From Fungi to Mycotrophic Orchid

**DOI:** 10.3389/fpls.2021.793876

**Published:** 2021-12-09

**Authors:** Jan Ponert, Jan Šoch, Stanislav Vosolsobě﻿﻿﻿, Klára Čiháková, Helena Lipavská

**Affiliations:** ^1^Department of Experimental Plant Biology, Faculty of Science, Charles University, Prague, Czechia; ^2^Prague Botanical Garden, Prague, Czechia; ^3^Institute of Botany, Czech Academy of Sciences, Průhonice, Czechia

**Keywords:** carbon transfer, histochemical localization, mycorrhiza, mycotrophy, orchid, carbohydrate, trehalase, trehalose

## Abstract

Orchids rely on mycorrhizal symbiosis, especially in the stage of mycoheterotrophic protocorms, which depend on carbon and energy supply from fungi. The transfer of carbon from fungi to orchids is well-documented, but the identity of compounds ensuring this transfer remains elusive. Some evidence has been obtained for the role of amino acids, but there is also vague and neglected evidence for the role of soluble carbohydrates, probably trehalose, which is an abundant fungal carbohydrate. We therefore focused on the possible role of trehalose in carbon and energy transfer. We investigated the common marsh orchid (*Dactylorhiza majalis*) and its symbiotic fungus *Ceratobasidium* sp. using a combination of cultivation approaches, high-performance liquid chromatography, application of a specific inhibitor of the enzyme trehalase, and histochemical localization of trehalase activity. We found that axenically grown orchid protocorms possess an efficient, trehalase-dependent, metabolic pathway for utilizing exogenous trehalose, which can be as good a source of carbon and energy as their major endogenous soluble carbohydrates. This is in contrast to non-orchid plants that cannot utilize trehalose to such an extent. In symbiotically grown protocorms and roots of adult orchids, trehalase activity was tightly colocalized with mycorrhizal structures indicating its pronounced role in the mycorrhizal interface. Inhibition of trehalase activity arrested the growth of both symbiotically grown protocorms and trehalose-supported axenic protocorms. Since trehalose constitutes only an inconsiderable part of the endogenous saccharide spectrum of orchids, degradation of fungal trehalose likely takes place in orchid mycorrhiza. Our results strongly support the neglected view of the fungal trehalose, or the glucose produced by its cleavage as compounds transported from fungi to orchids to ensure carbon and energy flow. Therefore, we suggest that not only amino acids, but also soluble carbohydrates are transported. We may propose that the soluble carbohydrates would be a better source of energy for plant metabolism than amino acids, which is partially supported by our finding of the essential role of trehalase.

## Introduction

Mycorrhizal symbiosis is a common phenomenon in almost all land plants (Smith and Read, 2008). Intensive research is focused mostly on mutualistic mycorrhizal symbioses, where plant carbon- and energy-containing compounds are exchanged with fungi for mineral nutrients (van der Heijden et al., 2015). However, mycorrhizal symbioses are not only mutualistic, but also parasitic, for example in mycoheterotrophic plants (Leake, 1994; Taylor et al., 2003; Merckx, 2013). Our knowledge on these one-sided relationships is scant compared to that about mutualistic mycorrhizal associations.

Orchids, of which there are more than 27,000 species (WCSP, 2020), are the largest and the most intensively studied group of mycoheterotrophic plants. All orchid species produce tiny seeds with little reserves, which develop into mycoheterotrophic protocorms (Arditti and Ghani, 2000; Rasmussen and Rasmussen, 2009; Rasmussen et al., 2015). The majority of orchids are initially mycotrophic and photosynthesize when mature, while only some species remain fully mycoheterotrophic for their whole life (Dearnaley et al., 2016; Selosse et al., 2016; Tĕšitel et al., 2018). Fungal hyphae form large coils called pelotons inside orchid cells, which are enveloped in the plant perifungal membrane (Hadley et al., 1971; Peterson et al., 1998; Bougoure et al., 2014). After pelotons reach their final size, they can stay in the cells for some time, but later they become senescent and are digested (Rasmussen, 1990; Peterson et al., 1998; Bougoure et al., 2014). It is not clear whether nutrient transfer occurs during the stage of the living peloton biotrophically (Mollison, 1943; Hadley and Williamson, 1971; Cameron et al., 2006, 2008) or necrotrophically during its degradation (Bernard, 1902; Hadley, 1984; Bougoure et al., 2014), and evidence exists for both ways (Kuga et al., 2014; Selosse, 2014).

Orchids can obtain several elements from mycorrhizal fungi, as demonstrated for carbon (Gebauer and Meyer, 2003; Trudell et al., 2003; Suetsugu and Matsubayashi, 2021), nitrogen (Gebauer and Meyer, 2003; Schiebold et al., 2017; Suetsugu and Matsubayashi, 2021; Valadares et al., 2021), phosphorus (Alexander et al., 1984), hydrogen (Schiebold et al., 2017; Schweiger et al., 2018), and oxygen (Yoder et al., 2000). Most of these elements can be transferred also in other types of mycorrhizal association. In contrast to mutualistic mycorrhizas of autotrophic plants, non-green plants are generally considered chemoheterotrophic, which means they obtain carbon and energy from exogenous organic compounds, specifically from fungi (Smith and Smith, 1990; Leake, 1994; Merckx, 2013). As the source of energy is hard to identify, many researchers focus on carbon transfer only. Detailed observation of ^13^C transfer using secondary ion mass spectrometry (SIMS) has indicated that more than a single compound is involved in carbon transfer (Kuga et al., 2014). However, the true identity of these compounds remains elusive. Two groups of compounds have been hypothesized to participate in carbon transfer in orchid mycorrhiza: amino acids (Cameron et al., 2006, 2008; Dearnaley and Cameron, 2017; Fochi et al., 2017) and soluble carbohydrates (Smith, 1967; Cameron et al., 2006).

Cameron et al. (2006, 2008) demonstrated that labeled carbon, provided to the fungus *Ceratobasidium cornigerum* in glycine or amino acid mixture, was transferred into the orchid *Goodyera repens* and hypothesized that amino acids could be involved in carbon transfer. Similar results were later obtained by Bougoure et al. (2010) with *Rhizanthella gardneri* and *Ceratobasidium* sp. Glutamine is regarded as the compound responsible for major nitrogen flow from ectomycorrhizal fungi to their host plants (Martin et al., 1987; Smith and Smith, 1990). Similar glutamine transport ensuring nitrogen flow perhaps operates also in orchid mycorrhiza, and at least some portion of C is supposed to be transported in the form of this amino acid (Dearnaley and Cameron, 2017; Fochi et al., 2017). However, the amino acids were probably at least partially metabolized by fungi because fungal respiration of labeled C was observed in studies on *G. repens* (Cameron et al., 2006, 2008), and the ^13^C:^15^N ratio in plant biomass was markedly different from that provided in glycine (Cameron et al., 2006; Bougoure et al., 2010). The elevated ^13^C:^15^N ratio observed in *G. repens* could be attributed to fungal transamination (Cameron et al., 2006); however, the ^13^C:^15^N ratio was lowered in *R. gardneri* (Bougoure et al., 2010), indicating more complex situation. The elevated values of the aforesaid ratio in *G. repens* might be the result of carbon transport in the form of some nitrogen-free compound (Cameron et al., 2006). The existence of such two carbon-containing compounds transported in parallel could explain the variation in the ^13^C:^15^N ratio between the two species investigated. In experiments with two *Anacamptis* species, ^13^C was transferred into orchids to a comparable extent when supplied to the fungus in the form of glucose or that of an amino acid mixture (Alghamdi, 2020). Taken altogether, the transport of amino acids alone cannot explain the obtained results, so another pathway for carbon transfer likely exists.

Soluble carbohydrates are the compounds widely used as energy and carbon sources in various symbiotic interactions ranging from parasitism (Talbot, 2010) to mutualism (Hampp & Schaeffer, 1999; Wiemken, 2007; Nehls et al., 2010). The first attempts to characterize carbon and energy transport into mycotrophic orchids therefore focused on soluble carbohydrates. Numerous articles point to the need of external soluble carbohydrate to support axenic growth of heterotrophic orchid life stages (see Arditti, 1967; Rasmussen, 1995 and references therein). Smith (1967) investigated carbon transfer from *Rhizoctonia* strains to protocorms of *Dactylorhiza purpurella* using ^14^C-labeled glucose. The fungus firstly incorporated ^14^C into trehalose, which was subsequently transported into mycorrhizal tissue and was finally found in plant-specific sugar sucrose. Based solely on the tracing of ^14^C, the author hypothesized that trehalose could be the fungal carbohydrate used for the synthesis of plant sucrose and therefore involved in the transport of carbon from the fungus to the orchid (Smith, 1967). The supposed transport of trehalose was discussed by Cameron et al. (2006) as a possible explanation of the elevated ^13^C:^15^N ratio transported into *G. repens*. The role of soluble carbohydrate transport in orchid mycorrhiza is also supported by the results of transcriptomic studies. It has been found that the expression of orchid genes encoding putative sugar transporters of the Sugars Will Eventually be Exported Transporters (SWEET) family are upregulated in mycorrhizas of *Serapias vomeracea* with *Tulasnella calospora* (Perotto et al., 2014), *Bletilla striata* with *Tulasnella* sp. (Miura et al., 2018), in mycorrhizal roots of *Limodorum abortivum* (Valadares et al., 2021), or in albino, fully mycotrophic individuals of the otherwise usually green orchid *Epipactis helleborine* (Suetsugu et al., 2017). From this point of view, the ability of orchids to grow on external trehalose, an abundant fungal soluble carbohydrate (e.g., Smith, 1967; Jorge et al., 1997), is of interest (Smith, 1973). Several orchid species have been reported to grow *in vitro* on trehalose as the sole sugar source (Ernst et al., 1971; Smith, 1973; Hadley and Purves, 1974; Jheng et al., 2006; Liu et al., 2006; Sopalun et al., 2010). These results were usually restricted to the evaluation of growth only and were not supported by endogenous metabolite analysis, so the comparison of trehalose with other soluble carbohydrates is only vague. Importantly, comparable concentrations of external trehalose are toxic for *Arabidopsis thaliana* (Wingler et al., 2000; Schluepmann et al., 2004; Delatte et al., 2011) and *Cuscuta reflexa* (Veluthambi et al., 1982). Higher plants are generally known to be sensitive to external trehalose (Paul et al., 2008) and modifications of their endogenous trehalose levels can also affect the levels of its precursor trehalose-6-phosphate (T6P; Wingler et al., 2000; Schluepmann et al., 2004; Delatte et al., 2011). This compound acts as a powerful signaling molecule (Ponnu et al, 2011 Tsai and Gazzarrini., 2014), so changes in endogenous levels of trehalose (Delorge et al., 2014), including those achieved by exogenous trehalose application (Wingler et al., 2000; Schluepmann et al., 2004; Delatte et al., 2011), are known to cause dramatic effects in plants. Thus, the unique ability of orchids to utilize trehalose rises an interesting question: Could this ability be directly related to mycotrophy (Smith, 1973)?

Parallel transfer of carbon in the form of both, soluble carbohydrates and amino acids seems possible. However, direct evidence is missing. Our objective was to investigate whether trehalose and its degradation could actually take place in orchid mycorrhiza. To clarify whether trehalose could serve as the main energy and carbon source for orchids, we performed a robust examination of the utilization of trehalose and other soluble carbohydrates by *Dactylorhiza majalis*. To determine whether trehalose degradation could occur in mycorrhizas, we performed histochemical localization of trehalase in mycorrhizal tissues. We also applied a trehalase-specific inhibitor to test the significance of trehalase operation for mycorrhizal interaction.

## Materials and Methods

### Plant Material

The ripe seeds were collected from plants established in garden (originated from the locality Rádlo, Czech Republic, GPS: 50.708N, 15.121E) and stored with silica gel at +15°C for maximum one year. Roots of *D. majalis* (Common Marsh Orchid) were collected from the same plants (established in a garden for 10years prior experiment).

### Fungal Material and Culture

*Ceratobasidium* sp. isolate, widely engaged for *Dactylorhiza in vitro* propagation (commonly under the name A36, provided by Hardy Orchid Society, Great Britain), was maintained by subculturing each 3months onto fresh OMA medium (powdered oat meals 3g·L^−1^, agar 10g·L^−1^; Perotto et al., 2014) and grown in dark at 20°C. To confirm fungus identity, the DNA was extracted with ZR Fungal/Bacterial DNA kit (Zymo Research, United States), two regions of rDNA were directly amplified using DreamTaq DNA Polymerase (following manufacturers instructions): ITS with primers ITS1F and ITS4 (Gardes and Bruns, 1993) and 18S with primers NS1 and NS24 (White et al., 1990; Gargas and Taylor, 1992). The PCR conditions were as follows: 1min of denaturation at 95°C, followed by 32cycles at 95°C for 30s, 20s at 54°C (ITS)/49°C (18S), 50s at 72°C, and a final extension of 7min at 72°C. Sequences were submitted to GenBank: ITS: KY014293, 18S: KY014294. Closest GenBank matches according to BLAST Maximum Score were: ITS: MH855685=*Rhizoctonia alpina* CBS 309.35, coverage 99%, similarity 98.9%; 18S: AY757266=*Ceratobasidium* sp. GEL5602, coverage 100%, similarity 99.0%.

### *In vitro* Plant Culture and Comparison of Soluble Carbohydrates

The seeds were sown by a technique described in Ponert et al. (2013). Briefly, the seeds were treated with 70% ethanol for 5min, washed 3 times with distilled water, treated with calcium hypochlorite solutions (20g dissolved in 100ml water, filtered, drop of Tween 20 added) for 5min, washed 3 times with sterile distilled water, and the suspension of seeds was poured onto solid medium in 9-cm Petri dishes. The dishes were sealed by air permeable foil (Parafilm M) and kept in dark at 23°C or 20°C for asymbiotic or symbiotic cultures, respectively.

Asymbiotic cultivations were performed on medium SMS without inositol (Ponert et al., 2013; Ponert and Lipavská, 2017) consisting exclusively of defined substances and thus enabling to control precisely soluble carbohydrate supply in experiments. D-carbohydrates were added prior autoclaving (100mM monosaccharides, 50mM disaccharides, 33mM raffinose). Soluble carbohydrate content determination in the autoclaved media (HPLC with refractometric detection) revealed 4.3% sucrose decomposition into glucose and fructose and no degradation detectable in other carbohydrate variants. The medium did not contain any other soluble carbohydrates. Aqueous solution of a trehalase inhibitor Validamycin A was filter sterilized and added into cooled medium after autoclaving to reach final concentration 150μM.

For symbiotic cultures, we adopted widely used technique described, e.g., in Perotto et al. (2014), seeds were sown on the same OMA medium as used for fungi maintenance, and approximately, 3×3mm piece of fungus-infected medium was placed on the medium in 9-cm Petri dish.

### Germination Rate and Protocorm Size Analysis

Asymbiotically grown orchid protocorms were analyzed 4months after sowing (4–6 plates from each treatment, 80–150 protocorms from each plate). Symbiotically grown protocorms grew faster, so they were analyzed at corresponding developmental stage 2months after sowing. Germination rate and protocorm size expressed as maximum protocorm diameter were determined as described in Ponert et al. (2013).

To test the effect of Validamycin A also on the older protocorms, which were not germinating in the presence of this inhibitor, we precultured protocorms symbiotically for 2months on OMA medium, transplant them on experimental variants (medium OMA with/without 150μM Validamycin A) and cultivated for next 2months prior analyses.

Differences in germination rate were statistically tested with ANOVA followed by the Tukey–Kramer test (Kramer, 1956). Differences in protocorm size were tested with a nested ANOVA followed by the Tukey–Kramer test (Faria et al., 2014). The factor of a Petri dish was nested within the factor of experimental variants. Log or sqrt transformation was applied on data without normal distribution prior the ANOVA analysis. Differences between only two categories were tested with Wilcoxon signed-rank test (Wilcoxon, 1945). Statistical analyses were performed in the R 3.1.2. statistical software package (R Core Team, 2019) at *α*=5% probability level.

### Fungal Growth Rate Analysis

Approximately, 3×3mm piece of fungus-inoculated medium was placed on the new OMA medium (with/without 150μm Validamycin A) in 9-cm Petri dish (seven plates per treatment). The colony diameter was measured daily to identify the phase of constant growth. The daily increase in colony radius was then calculated as a mean from days 3, 4 and 5 after inoculation, when the growth was constant in both experimental variants. Difference was tested with Wilcoxon signed-rank test (Wilcoxon, 1945).

### Carbohydrate Content Analysis

Protocorms were carefully collected from the surface of the medium using a combination of different tweezers to avoid damaging the medium and to minimize the possibility of contamination with the medium. Protocorms from each Petri dish were analyzed separately. Soluble carbohydrates were extracted in aqueous solution as described (Ponert and Lipavská, 2017) and analyzed using an HPLC system with refractometric detection (column 250 × 8mm filled with IEX Pb form 8μm; Watrex, Czech Republic) following the protocol of Vojtíšková et al. (2006).

The pellets after soluble carbohydrate extraction were used for starch analysis. Starch was enzymatically degraded by α-amylase and amyloglucosidase (Steinbachová-Vojtíšková et al., 2006), and glucose was quantified with the HPLC system described above, except for the use of IEX Ca form 8μm column in this case.

To extract soluble carbohydrates from media, 0.5g of medium was mixed with 0.5ml ultrapure water, incubated at +4°C for 5h, centrifuged for 10min and filtered (0.22μm membrane filter).

As the HPLC system described above does not allow separation of sucrose and trehalose, the soluble carbohydrate samples from experiment with trehalase inhibitor Validamycin A were further analyzed using high-performance anion exchange chromatography with pulsed amperometric detection (HPAEC-PAD) and CarboPac™ PA10 column. Sucrose and trehalose contents were quantified following the ICUMSA Method GS7/4/8–24 (ICUMSA, 2011), and the sucrose-to-trehalose ratio in each sample was calculated. Data were statistically analyzed as described for protocorm size.

### Histochemical Localization of Trehalase

Roots collected in May were sectioned to 2–3cm long sections and immediately cut into slices (20–40μm width) using a hand microtome. The protocorms from *in vitro* cultivations were sectioned longitudinally in the middle with a razor blade. The sections were cut under a fixative solution (2% paraformaldehyde, 2% polyvinylpyrrolidone PVP-40, 5mM 1,4-dithiothreitol; Doehlert and Felker, 1987), immediately transferred into a vial with cooled fixative solution and incubated on ice for 1h. The sections were washed 3 times with cooled sodium–phosphate buffer (0.38M, pH 6.0), placed in the same buffer for 12h at 2–4°C. (Buffer was exchanged 7 times during this time to completely remove soluble carbohydrates.) The reaction mixture (0.24mgml^−1^ nitro blue tetrazolium, 0.14mgml^−1^ phenazine methosulfate, 25Uml^−1^ glucose oxidase, 10mgml^−1^ trehalose, 9μM diphenyleneiodonium chloride, in 0.38M sodium–phosphate buffer pH 6.0) was added, and the samples were incubated in dark at 30°C on a rotary shaker (100 RPM). The addition of trehalose to the sections with trehalase resulted in production of glucose, which was visualized by coupled reactions resulting in production of blue precipitate formazan (Dalhlqvist and Brun, 1962 Doehlert and Felker, 1987). Reaction without trehalose was used as a negative control. Reactions were checked regularly and stopped when blue coloration was visible or after 22h. The sections were washed 3 times with the sodium–phosphate buffer and stored in this buffer at 2–4°C in the dark until observation (no longer than 12h). Images were taken using a digital camera (Canon EOS 60D) mounted on a stereomicroscope Olympus SZ X7 or on a microscope Olympus Provis AX70 for higher magnification.

## Results

### Trehalose Is Readily Utilized by Orchid Protocorms

**Figure 1:**
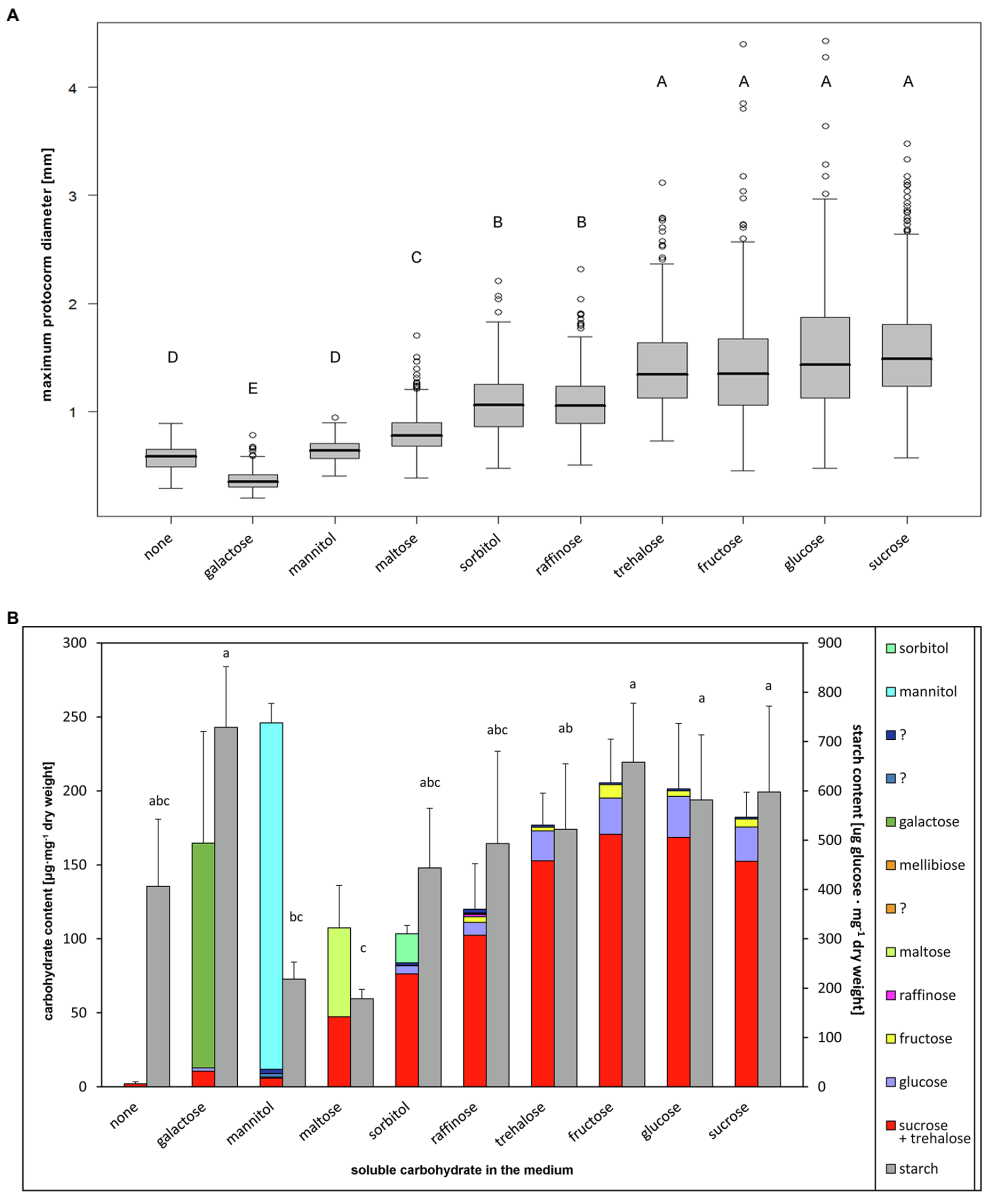
Effect of soluble carbohydrate type in the medium on *Dactylorhiza majalis* protocorms. 4-month cultivation on medium SMS with 100mM monosaccharides, 50mM disaccharides, 33.3mM raffinose. **(A)** Protocorm size, median values are given. Nested ANOVA followed by TukeyC test (log-transformed data, *α*=0.05). **(B)** Non-structural carbohydrate contents in protocorms, mean values and SD of total soluble carbohydrate and starch contents are given. Different letters indicate significant differences according to ANOVA followed by the Tukey–Kramer test (*α*=0.05). For soluble carbohydrate statistics see Supplementary Table 1.

To compare trehalose ability to serve as energy and carbon source with other soluble carbohydrates, we evaluated growth and endogenous non-structural carbohydrate levels of protocorms, which were cultivated asymbiotically on media with different soluble carbohydrates. Protocorm size differed significantly between treatments (Nested ANOVA on log transformed data, treatment effect: *F*_[9, 32]_=182.4, *p*≤2×10^−16^). On the control medium without soluble carbohydrates, seeds germinated, protocorms developed rhizoids and stopped growth at very small size (Figure 1A; Supplementary Figures 1, 2), and only small amount of sucrose was detectable in their tissues (Figure 1B; Supplementary Table 1). The ability of protocorms to utilize each of the tested soluble carbohydrates varied considerably, allowing us to divide these carbohydrates into five groups (Figure 1; Supplementary Figure 1; Supplementary Table 1):

(i) Galactose—toxic, killing embryos in the seeds at very early stages, causing development of dark brown necrotic embryos, which did not produce hairs (Figure 1; Supplementary Figure 1), with very low endogenous non-structural carbohydrates levels; (ii) Mannitol—not lethal for embryos or protocorms but stimulating only very weak non-significant protocorm growth (Figure 1A; Supplementary Figure 1). Interestingly, the protocorms accumulated mannitol substantially, though they appear to have utilized it only at imperceptible level; (Figure 1B; Supplementary Table 1); (iii) Maltose—utilized by protocorms to some extent, slightly supporting protocorm growth (Figure 1A; Supplementary Figure 1) and accumulating in protocorm tissues to represent about a half of the endogenous sugar content (Figure 1B; Supplementary Table 1); (iv) Sorbitol and raffinose—utilized by protocorms, supporting protocorm growth rather well, however, still at significantly lower degree than readily utilizable carbohydrates as sucrose (Figure 1A; Supplementary Figure 1). Protocorms contained higher levels of endogenous soluble carbohydrates with sucrose being dominant in the spectrum. Only low levels of sorbitol and raffinose (in sorbitol and raffinose supported protocorms, respectively) were present in tissues, indicating a sufficient utilization rate (Figure 1B; Supplementary Table 1); (v) Fructose, glucose, sucrose, and trehalose—readily utilized by protocorms. These sugars supported protocorm growth at a comparable level (Figure 1A; Supplementary Figure 1). Protocorms contained high levels of sucrose, glucose, and fructose, with sucrose strongly prevailing in the spectrum, and the contents of individual endogenous sugars were also comparable with the exception of endogenous fructose (Figure 1B; Supplementary Table 1).

### Trehalose Utilization Is Sensitive to Trehalase Specific Inhibitor Validamycin A

**Figure 2:**
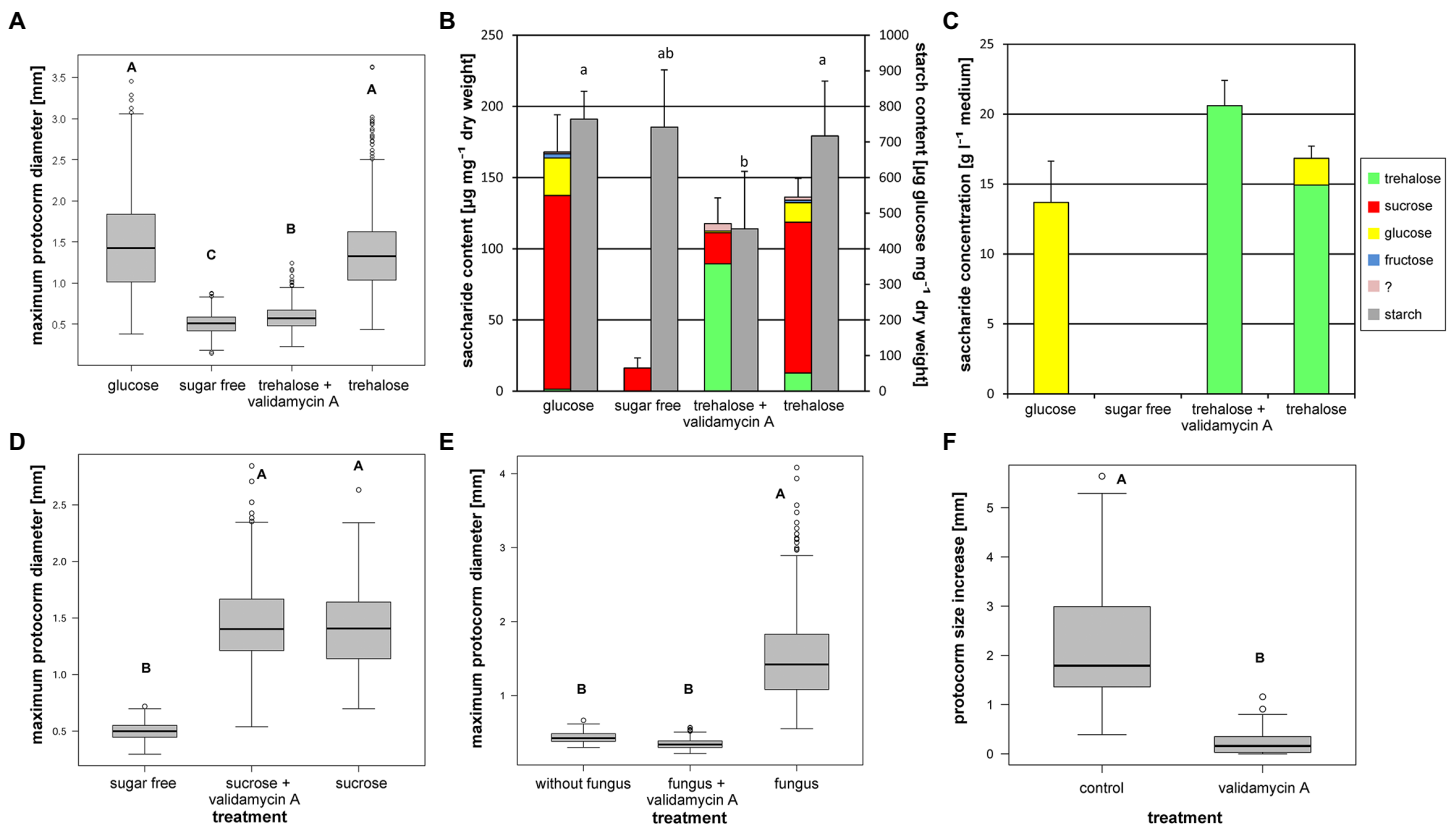
Effect of trehalase specific inhibitor Validamycin A on *D. majalis* protocorm characteristics. Cultivation on symbiotic medium OMA or asymbiotic medium SMS with 100mM glucose or 50mM disaccharides; modified to contain 0 or 150μM Validamycin A. Different letters indicate statistically significant differences. **(A)** Validamycin A inhibits growth of trehalose-supported protocorms (4-month cultivation, medians, Nested ANOVA followed by TukeyC test on log-transformed data). **(B)** Non-structural carbohydrate contents in protocorms from (A) (mean values and SD of total soluble carbohydrate and starch contents, ANOVA, Tukey–Kramer test. For statistics see Supplementary Table 2). **(C)** Soluble carbohydrate contents in the media after cultivation of protocorms from (A) (mean values and SD of total soluble carbohydrate content, for statistics see Supplementary Table 3) **(D)** Validamycin A has no effect on sucrose-supported protocorms (4-month cultivation, medians, Nested ANOVA followed by TukeyC test on log-transformed data). **(E)** Validamycin A blocks growth of symbiotic protocorms grown from seeds directly on experimental media (2-month cultivation, medians, Nested ANOVA followed by TukeyC test on log-transformed data). **(F)** Validamycin A inhibits growth of older symbiotic protocorms (precultured for 2months without the inhibitor, measured increase in size during subsequent culture in experimental conditions next 2months, Wilcoxon signed-rank test).

To obtain some evidence for expected trehalase involvement in observed trehalose utilization, we applied trehalase specific inhibitor, Validamycin A, on asymbiotic *in vitro* protocorm cultures. The inhibitor did not affect sucrose-supported cultures at all (Nested ANOVA on log transformed data, treatment effect: *F*_[2, 12]_=736.5, *p*<2.8×10^−13^; Figure 2D), while significantly restricted growth of trehalose-supported protocorms (Nested ANOVA on log transformed data, treatment effect: *F*_[3, 25]_=292.8, *p*≤2×10^−16^; Figure 2A). Trehalose-supported, Validamycin-A-treated protocorms exhibited similarly negligible growth as protocorms cultivated in the absence of soluble carbohydrates in the medium (Figure 2A).

The Validamycin-A-treated protocorms also accumulated trehalose at significantly higher levels than the non-inhibited ones, they contained no detectable glucose, and they exhibited significantly lower levels of sucrose (Figure 2B) comparable with that of the protocorms cultivated in the absence of soluble carbohydrates in the medium (Supplementary Table 2). The cultivation medium of trehalose-supported variant contained significant amount of glucose (Figure 2C; Supplementary Table 3). In the Validamycin-A-supplemented trehalose medium, glucose was not detectable, and trehalose content was significantly higher (Figure 2C; Supplementary Table 3).

### Trehalase Activity Colocalizes With Living Fungal Structures in Mycorrhizas

**Figure 3:**
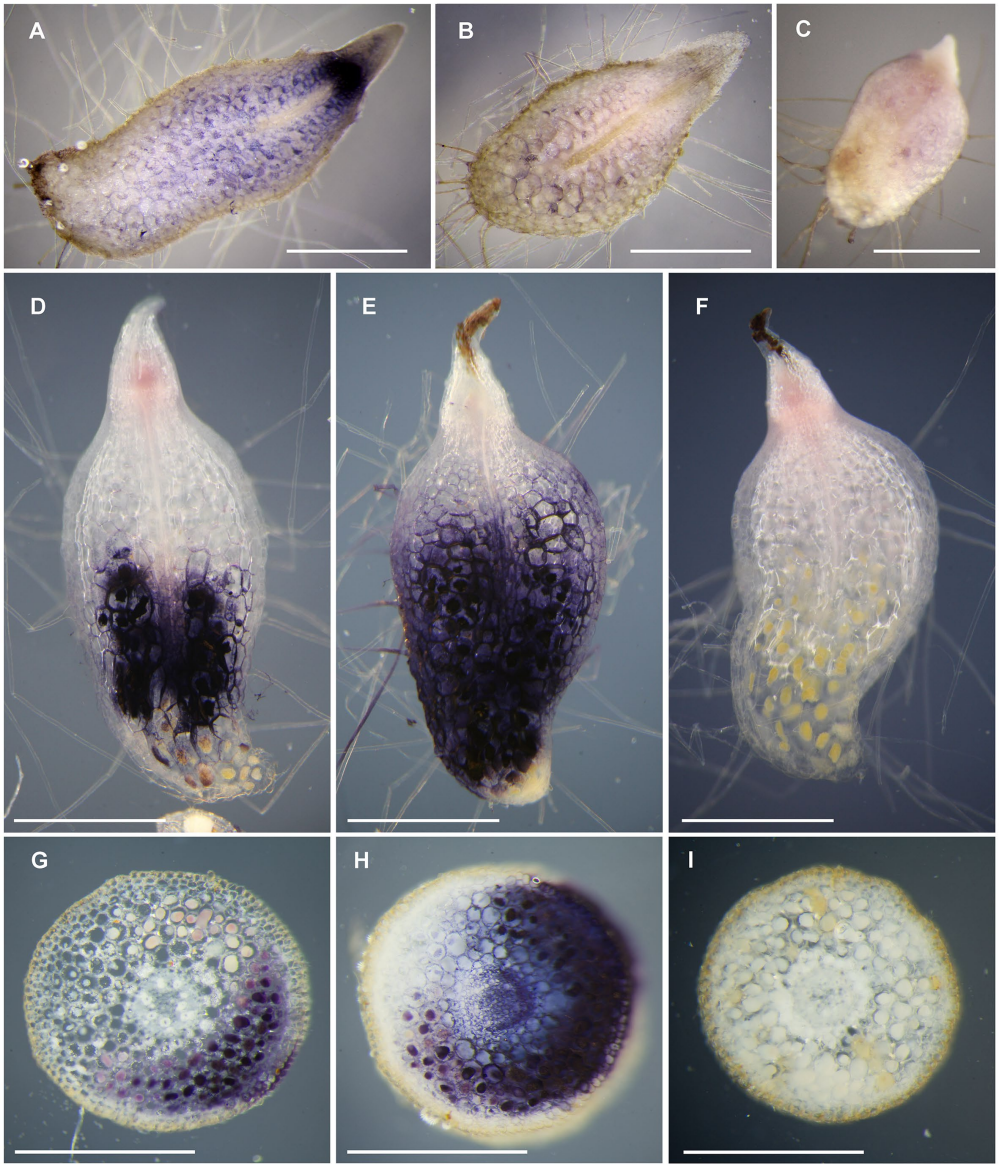
Histochemical detection of trehalase activity in *D. majalis* tissues. Blue colored formazan precipitate marks trehalase activity. Control incubations without added substrate did not stain **(C,F,I)**. **(A,C)** Longitudinal sections of trehalose-supported asymbiotic protocorms, **(B)** glucose-supported asymbiotic protocorm, and **(D-F)** symbiotic protocorms cultured with *Ceratobasidium* sp. **(G-I)** cross sections of mycorrhizal roots of mature plants. The trehalase activity is present in the vicinity of pelotons **(D,E,G,H)**. Scale bars 1mm.

To characterize spatial distribution of trehalase in orchid mycorrhizal and non-mycorrhizal tissues, we performed histochemical localization of trehalase activity. In asymbiotic, trehalose-supported protocorms, trehalase activity was present in the whole tissue with the highest activity in/around meristematic pole (Figure 3A). The time to staining was relatively long (6.3±0.5h, Supplementary Table 4). After an extremely long time of staining (22h), when trehalose-supported protocorms were heavily over-stained, weak trehalase activity was detectable in sucrose- and glucose-supported protocorms. However, the staining was very faint (Figure 3B), so we classified these sections as unstained in Supplementary Table 4. Control reactions without trehalose were completely free of detectable staining, even after 22h (Figure 3).

Contrastingly, in symbiotic protocorms, time to staining was short (0.5h, Supplementary Table 4), and trehalase activity was localized in basal protocorm part, where mycorrhizal structures (pelotons) occurred (Figures 3D,E). Only in two out of 48 protocorms, staining excessed mycorrhizal part and reached the apical protocorm part. In majority of protocorms (31 out of 48), the basalmost pole lacked trehalase activity (Figure 3D). Only old senescent pelotons were present in these unstained parts in all cases.

In mycorrhizal roots, time to staining was short (0.5h; Supplementary Table 4), and the activity was present only in the sections, where some pelotons occurred. Within the section, activity was detectable in the cells with pelotons and usually also in a few neighboring cells (Figures 3G-I). Areas distant from all pelotons were free of trehalase activity in all cases (Figure 3H). In ten sections out of 50, unstained areas with pelotons and without activity were observed (Figure 3G).

### Inhibition of Trehalose Utilization by Validamycin A Stops Growth of Mycotrophic Protocorms

To test expected trehalase involvement in carbon transfer in mycorrhiza, we applied trehalase-specific inhibitor, Validamycin A, on symbiotic *in vitro* cultures. In the pure fungal culture on OMA medium, the inhibitor reduces fungus growth rate (*p*=0.0012) but still enables growth. Mean daily fungal growth rate without the inhibitor was 3.57±0.43mm, while 0.93±0.29mm in the presence of the inhibitor. The whole agar plate was covered with mycelium after 6–9days in control variants and after 14 to 30days in Validamycin-A-treated variants.

In a symbiotic culture established by direct seed sowing on experimental medium, protocorm growth was completely blocked by Validamycin A (Nested ANOVA on log transformed data, treatment effect: *F*_[2, 12]_=471.1, *p*≤3.9×10^−12^; Figure 2E). Similarly, in symbiotic culture established by transplanting of precultured protocorms, the growth was strongly inhibited by Validamycin A (*p*≤2.2×10^−16^; Figure 2F).

## Discussion

### Trehalose Is a Readily Utilizable Carbon and Energy Source for Protocorms

The ability of trehalose to support growth has been reported for several species of orchids (Ernst et al., 1971; Smith, 1973; Hadley and Purves, 1974; Jheng et al., 2006; Liu et al., 2006; Sopalun et al., 2010). To test whether trehalose could really be an equivalent energy and carbon source to readily metabolizable carbohydrates as glucose or sucrose, we tested the ability of *D. majalis* to utilize a broad range of soluble carbohydrates. Four soluble carbohydrates supported the growth best, yielded the largest protocorms, and proved to be comparable in their effects, namely glucose, fructose, sucrose, and trehalose. The suitability of glucose, fructose, and sucrose for supporting protocorm growth has also been reported for various other orchid species (e.g., Burgeff, 1936; Arditti, 1967; Ernst, 1967; Wotavová-Novotná et al., 2007; Stewart and Kane, 2010). Our results show that endogenous non-structural carbohydrate spectra of asymbiotic protocorms grown on all four sugars are very similar, characterized by significant reserves of starch together with high levels of endogenous sucrose. The minor effect of exogenous trehalose on the endogenous non-structural carbohydrate spectrum in *D. majalis* protocorms is surprising. In non-orchid plants, dramatic changes in the endogenous non-structural carbohydrate spectrum have been observed after exogenous trehalose application (Müller et al., 1995; Best et al., 2011). We may conclude that *D. majalis* protocorms possess an efficient metabolic capability for the uptake and utilization of exogenous trehalose, which can serve as an energy source for orchid protocorms to the same extent as their prominent endogenous soluble carbohydrates.

Other soluble carbohydrates, however, differ in their effects. Galactose is toxic for *D. majalis* embryos, which corresponds well with the effects observed in other orchid species (Quednow, 1930; Wynd, 1933; Ernst, 1967; Ernst et al., 1971; Ponert and Lipavská, 2017). Surprisingly, the seeds cultivated on medium with galactose contained large amount of starch. There was also a relatively large amount of starch in the protocorms on the medium without soluble carbohydrates, and it is therefore possible that this starch is derived from seed reserves. The dormant seeds of *D. majalis* contain lipid and protein reserves; however, starch is produced during germination when the reserves are hydrolyzed (Rasmussen, 1990). Mannitol is probably not utilized by *D. majalis*. An inability to grow on mannitol has been observed in several other orchid species (Ernst, 1967; Smith, 1973; Purves and Hadley, 1976; Van Waes, 1984; Rasmussen, 1995; Johnson and Kane, 2013; Ponert and Lipavská, 2017) but *Galeola septentrionalis* (Nakamura, 1982) and *Phalaenopsis* hybr. (Ernst, 1967) are able to utilize mannitol to some extent. Maltose was utilized by protocorms only at a low rate in our experiments. Similarly, *Ophrys iricolor* subsp. *lojaconoi* and *Oeceoclades decaryana* grew only poorly on maltose (Ponert and Lipavská, 2017). Contrastingly, other orchid species tested (all from the subfamily Epidendroideae) were able to readily grow on maltose (Ernst, 1967; Ernst et al., 1971; Rahman et al., 2005; Sopalun et al., 2010). Sorbitol and raffinose were utilized by *D. majalis*; however, its protocorms grew significantly slower than on readily utilizable soluble carbohydrates though still better than on maltose. Similarly to our results, raffinose supported growth of the three other orchid taxa only weakly (Ernst, 1967; Ernst et al., 1971; Ponert and Lipavská, 2017), but *Ophrys iricolor* subsp. *lojaconoi* grew relatively well on it (Ponert and Lipavská, 2017). Sorbitol, by contrast, was found to exhibit various effects on different orchid species, ranging from an extensive growth (Ernst, 1967; Nakamura, 1982; Rahman et al., 2005) to (nearly) no growth stimulation (Johnson and Kane, 2013; Ponert and Lipavská, 2017).

We may conclude that the ability of orchids to utilize different soluble carbohydrates varies between taxa; however, the ability to utilize trehalose alongside glucose, fructose and sucrose may be conserved among them. Given that trehalose is a common and abundant carbohydrate in fungi (e.g., Smith, 1967; Jorge et al., 1997), it can be assumed that this unique ability of orchids is an adaptation to mycotrophy (Smith, 1973). It could also be argued that this could be an adaptation to heterotrophy in general, but the obligatory parasitic plant *C. reflexa* is sensitive to external trehalose (Veluthambi et al., 1982), so the unique ability of orchids to utilize high concentrations of external trehalose is likely related to mycotrophy rather than heterotrophy in general. Another carbohydrate commonly found among the main fungal endogenous soluble carbohydrates is mannitol (Lewis and Smith, 1967; Smith, 1967; Stribley and Read, 1974; Söderström et al., 1988; Martin et al., 1998; Koide et al., 2000; Tibbett et al., 2002). However, it did not support protocorm growth and was not utilized in our study. It is therefore likely not usable by orchids as a fungal source of carbon and energy.

### Trehalose Hydrolysis Can Occur Directly in Mycorrhizas

More than fifty years ago, Smith (1967) investigated symbiotic protocorms of *Dactylorhiza purpurella* and hypothesized that trehalose could be the fungal soluble carbohydrate involved in transport from fungi to orchids. If this was the case, we would expect rapid trehalose utilization close to the mycorrhizal interface between the orchid and the fungus. Trehalose degradation in both plants and fungi is mediated by the enzyme trehalase (Jorge et al., 1997; Lunn et al., 2014), so we expected high activity of this enzyme in mycorrhizal tissues. To test this assumption, we applied a method for the histochemical localization of trehalase activity. Because intermediates in coupled reactions are soluble and the final product formazan precipitates non-homogeneously within plant tissues, we were able to localize the activity at the scale of a few cells, similarly to other methods for the histochemical localization of other enzymes of carbohydrate metabolism (Doehlert and Felker, 1987; Miller and Chourey, 1992; Wittich and Vreugdenhil, 1998; Sayago et al., 2008; Ogawa et al., 2009). Trehalase activity appeared to be colocalized with fungal pelotons, even within a single root or protocorm section. Except for peloton-containing cells, it was sometimes present in closely neighboring cells. Trehalose degradation therefore seems to be localized directly in mycorrhizas, supporting the hypothesis of Smith (1967).

However, it is unclear whether the trehalase is produced by the fungus, by the orchid or by both counterparts. Trehalose is a common fungal endogenous soluble carbohydrate, and fungi possess efficient pathways for its degradation with trehalases (Jorge et al., 1997). It could therefore be expected that fungal enzymes perform the trehalose breakdown in mycorrhiza. However, our results obtained with asymbiotic cultures show that *D. majalis* also possesses its own efficient enzymatic apparatus for utilizing trehalose. This enzymatic apparatus is likely to involve also a trehalase, as it is the only plant enzyme currently known capable of cleaving trehalose (Lunn et al., 2014). To test the role of trehalase in trehalose utilization by protocorms, we applied the highly specific trehalase inhibitor Validamycin A (Asano et al., 1987; Kameda et al., 1987; Goddijn et al., 1997) to asymbiotic cultures. It had no effect on sucrose-supported protocorms, but it blocked the growth of trehalose-supported ones, indicating a specific role of plant trehalase in trehalose utilization by protocorms. Trehalase activity is probably upregulated by exogenous trehalose, as evidenced by strong trehalase activity detectable in asymbiotic trehalose-supported protocorms, and only very weak activity present in sucrose- or glucose-supported ones. All in all, both symbiotic organisms are able to maintain efficient trehalase activity and the question regarding the origin of trehalase in mycorrhiza therefore remains open.

### Trehalase Action Is Essential for Orchid Mycotrophy

The way in which carbon and other nutrients are transferred from fungi to orchids has been debated for more than 100years (Bernard, 1902; Selosse et al., 2011). The persistent but fundamental question is which compounds are transferred. Two groups of compounds have been hypothesized to play this role: amino acids and soluble carbohydrates.

Amino acids play a significant role in nitrogen transfer (Dearnaley and Cameron, 2017; Fochi et al., 2017). However, there is no clear evidence that amino acids are the main carbon source (Cameron et al., 2006, 2008). It should be noted that non-green heterotrophic plants, such as orchid protocorms, are dependent on fungi for acquiring not only carbon, but also energy. Soluble carbohydrates are much more promising candidates for mediators in energy transfer than amino acids, which are relatively poor energy sources for plant metabolism (Hildebrandt et al., 2015). Such a principle partially operates in ectomycorrhizas and arbuscular mycorrhizas, where carbon and energy move between symbionts in the opposite direction (Smith and Smith, 1990). This carbon flow is mediated by the transfer of fatty acids and soluble carbohydrates (Luginbuehl et al., 2017). Regarding the transport of soluble carbohydrates, plant sucrose is hydrolyzed into hexoses, which are subsequently used by mycorrhizal fungi to synthesize trehalose or other carbohydrates characteristic for fungi. Fungal synthesis of fungal-specific carbohydrates like trehalose perhaps creates a strong sink, driving the movement of glucose at the interface (Shachar-Hill et al., 1995; López et al., 2007; Wiemken, 2007; Nehls, 2008). Hydrolysis of fungal trehalose into glucose, which is then used by orchids to synthesize plant-specific sucrose and starch, would therefore represent an analogous situation offering enough carbon and energy to orchid protocorms.

To test the possible role of trehalose in carbon and energy transfer, we inhibited the enzyme trehalase by its specific inhibitor Validamycin A in symbiotic cultures. Trehalase inhibition resulted in nearly complete inhibition of protocorm growth, similarly as in trehalose-supported asymbiotic protocorms. We were unable to distinguish between its effects on fungal and plant trehalases. However, the inhibitory effect of Validamycin A was only weak in the axenic fungal culture and the fungus was still able to overgrow the whole plate relatively quickly. We may therefore expect that the main effect causing the protocorm growth inhibition occurred at the mycorrhizal interface and particularly impacted the relationship between symbionts, rather than the symbionts themselves. Important role of soluble carbohydrate utilization in orchid mycorrhizal symbiosis could also be indicated by the results of a proteomic study of *Oncidium sphacelatum* protocorms with *Ceratobasidium* sp., which revealed more intensive glycolysis and a more vigorous Krebs cycle, likely because of catabolism of an organic carbon source (Valadares et al., 2014). This could have been caused by catabolism of both amino acids and soluble carbohydrates, but amino acid catabolism requires more energy and is therefore less efficient than carbohydrate catabolism. If glucose is produced from fungal trehalose by trehalase, it is likely be used not only for direct sucrose synthesis, but also for catabolism in glycolysis and the Krebs cycle, in accordance with the results of Valadares et al. (2014).

Considering our results in the context of the above-mentioned literature data, we suppose that the degradation of fungal trehalose by trehalase at the mycorrhizal interface provides glucose to fuel orchid carbohydrate synthesis and meet energy and carbon demands. If this is the case, carbon gets transported from fungi to orchids in two ways—in the form of amino acids and in that of soluble carbohydrates. The observations of Kuga et al. (2014) indicate that more than one compound could be involved in the transfer of carbon from fungi to orchids, supporting the view that both amino acids and trehalose might be involved in the transfer of carbon.

### Orchid Trehalase Seems to Operate Extracellularly

Trehalose influences the critical trehalose-6-phosphate (T6P) signaling pathway inside plant tissues (Delatte et al., 2011; Lunn et al., 2014; Tsai and Gazzarrini, 2014), so higher plants in general are considered highly sensitive to external trehalose (Veluthambi et al., 1982; Wingler et al., 2000; Schluepmann et al., 2004; Paul et al., 2008; Delatte et al., 2011). One interesting question is how orchids can cope with the high levels of exogenous trehalose. A metabolomic study of the orchid *Phalaenopsis* “Edessa” show that endogenous levels of T6P and sucrose do not correlate with each other, which may suggest a different function of T6P signaling in orchids (Ceusters et al., 2019). However, this observation can also be explained by other mechanisms, such as sucrose storage in the vacuole, leaving open the possibility of normally functioning T6P signaling in orchids (Ceusters et al., 2019).

Trehalose could be cleaved extra- or intracellularly. In the first case, intracellular concentration of trehalose could remain unaltered, which would protect the trehalose-6-phosphate signaling pathway. The histochemical method used is unable to localize trehalase at the intracellular scale. However, some indirect evidence can be found in other results. We observed trehalase activity distributed throughout the tissue of asymbiotic protocorms but localized in the basal mycorrhizal part of symbiotic protocorms. Its activity in asymbiotic protocorms was considerably lower compared to mycorrhizal tissues, as revealed by marked differences in the time to staining. It could be argued that if symbiotic protocorms grew faster than asymbiotic ones, the enhanced trehalase activity, induced by the presence of a fungus, should be responsible. However, the difference in the level of activity is roughly 13-fold, whereas the difference in growth rate is only about 1.5-fold. We observed some trehalase-dependent trehalose hydrolysis (sensitive to Validamycin A) also in the medium after asymbiotic cultivation. We may hypothesize that in the case of asymbiotic protocorms, extracellular activity might be responsible for the hydrolysis of the majority of trehalose and that the histochemically detected activity inside protocorm tissues might hydrolyze only residual trehalose. In this case, similar levels of trehalase activity could be present also in non-colonized cells of symbiotic protocorms, but we would be unable to detect this activity by our method because of overstaining which spreads from mycorrhizal structures. Thus, the difference in distribution of trehalase activity between asymbiotic and symbiotic protocorms should be interpreted with caution.

Trehalose hydrolysis in the medium was observed, in *D. purpurella* protocorms, also by Ernst (1967), who speculated that the glucose level in the trehalose-containing medium was too low to satisfy the energy demands of protocorms. Ernst (1967) did not consider the possibility of rapid uptake and utilization of glucose by protocorms, which could lead to the same result (Smith, 1973). In that case, trehalase should be situated mostly on the outer side of the plasmatic membrane and resulting glucose should be immediately transported into the cytoplasm with only small losses into the cultivation medium. The only plant trehalase with known cellular localization, that of *A. thaliana*, is situated on the apoplastic side of the plasma membrane (Frison et al., 2007). The genes encoding putative sugar transporters of the SWEET family were found to be upregulated in mycorrhizas of various orchid species (Perotto et al., 2014; Suetsugu et al., 2017; Miura et al., 2018; Valadares et al., 2021). We may therefore suppose similar localization of orchid trehalase on the apoplastic side of the plasmatic membrane, coupled with glucose transport by the transporters of the SWEET family. Such arrangement would be advantageous for orchids. Fungi generally take up glucose efficiently (Shachar-Hill et al., 1995; Nehls, 2008), so free trehalase activity in the interfacial matrix could lead to significant competition over the uptake of produced glucose between the fungus and the plant. Apoplastic cleavage of trehalose would also effectively protect the intracellular trehalose pool and the associated T6P signaling pathway. However, further research will be necessary to determine the exact location where trehalose degradation takes place.

### Conclusion

The way how carbon gets transferred to mycoheterotrophic plants has been debated for a long time. We focused on the neglected hypothetical involvement of trehalose in the transfer of carbon and energy to the orchid *D. majalis* and found strong support for this hypothesis. We propose that fungal trehalose is cleaved by trehalase at the mycorrhizal interface and that the resulting glucose gets transported into orchid cells. However, further research will be necessary to clearly localize trehalase in mycorrhizae and to determine its origin. In the context of other results, two groups of compounds likely secure carbon transfer in orchid mycorrhizas: amino acids and soluble carbohydrates.

## Data Availability Statement

The datasets presented in this study can be found in online repositories. The names of the repository/repositories and accession number(s) can be found at: https://www.ncbi.nlm.nih.gov/genbank/, KY014293, KY014294.

## Author Contributions

JP, JS, and HL designed the study and drafted the manuscript. JP, JS, SV, and KC performed experiments and analyzed the data. All authors contributed to the article and approved the submitted version.

## Funding

This work was financed by the Grant Agency of Charles University (project no. 924516). Additional support was provided as part of a long-term research project of the Czech Academy of Sciences, Institute of Botany (RVO 67985939).

## Conflict of Interest

The authors declare that the research was conducted in the absence of any commercial or financial relationships that could be construed as a potential conflict of interest.

## Publisher’s Note

All claims expressed in this article are solely those of the authors and do not necessarily represent those of their affiliated organizations, or those of the publisher, the editors and the reviewers. Any product that may be evaluated in this article, or claim that may be made by its manufacturer, is not guaranteed or endorsed by the publisher.
