# Supplementary material for: Integrative Study Supports the Role of Trehalose in Carbon Transfer From Fungi to Mycotrophic Orchid
Source: Front Plant Sci. 2021 Dec 9;12:793876. doi: 10.3389/fpls.2021.793876 (PMC8695678; doi:10.3389/fpls.2021.793876)
Supplement: Supplementary file 1 [file Data_Sheet_1.docx]

Supplementary Material


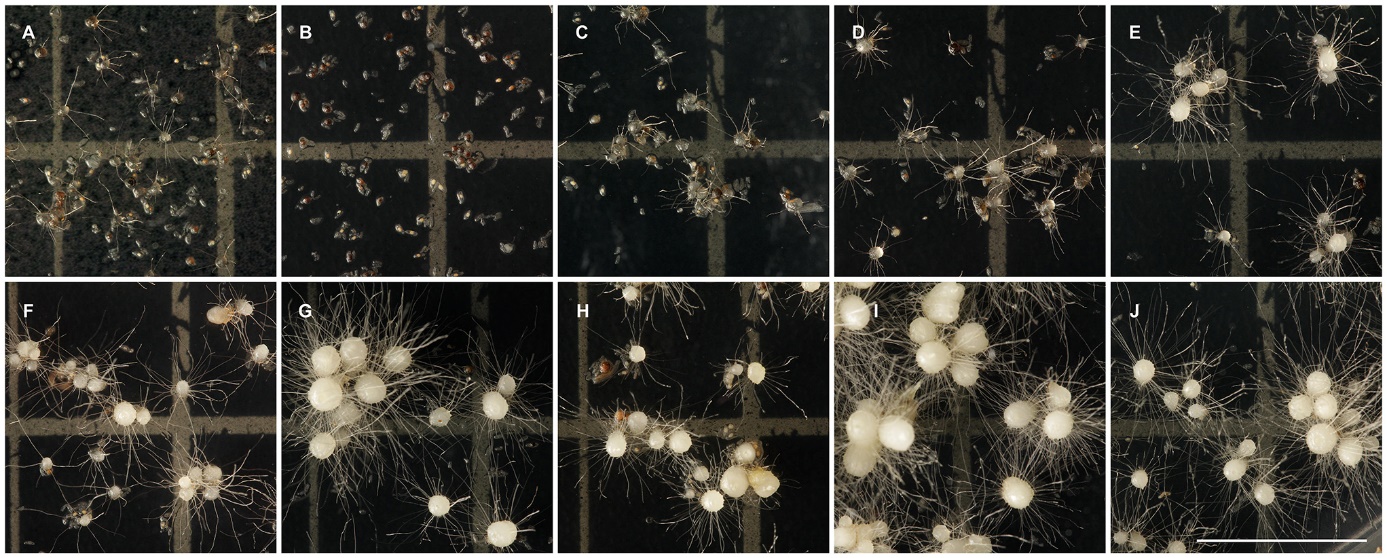


**Supplementary Figure 1.** Representative photographs of *D. majalis* protocorms grown on SMS medium with different soluble carbohydrates. (A) Without saccharide, (B) Galactose, (C) Mannitol, (D) Maltose, (E) Sorbitol, (F) Raffinose, (G) Trehalose, (H) Fructose, (I) Glucose, (J) Sucrose. 100 mM monosaccharides, 50 mM disaccharides, 33.3 mM raffinose Scale bar 1 cm (all pictures are in the same scale).


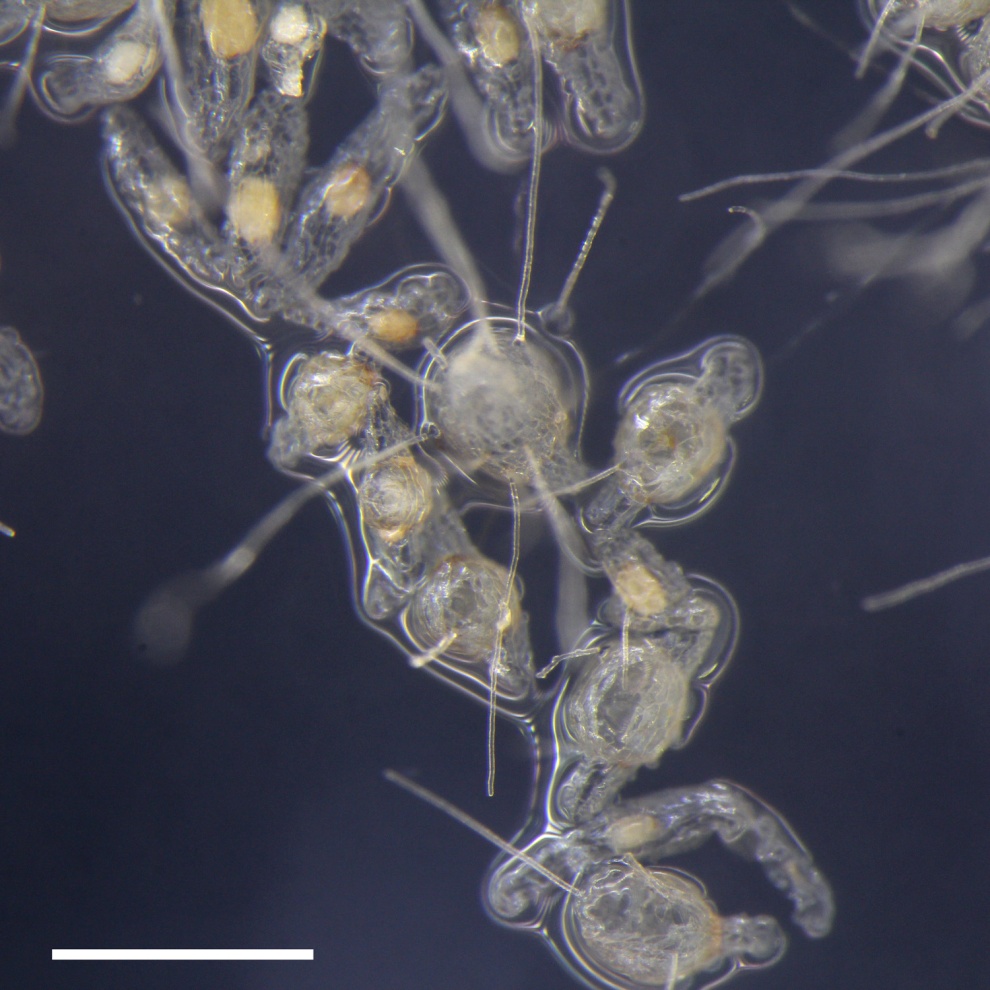


**Supplementary Figure 2.** *Dactylorhiza majalis* protocorms after 4-months cultivation on soluble carbohydrate free medium. Protocorms stopped growth early after germination. Scale bar 1 mm.

| **Carbohy-drate detected**  **[μg mg^-1^ dry weight]** | **soluble carbohydrate in the medium** | | | | | | | | | | **statistics** | | |
| --- | --- | --- | --- | --- | --- | --- | --- | --- | --- | --- | --- | --- | --- |
|  | **none** | **galactose** | **mannitol** | **maltose** | **sorbitol** | **raffinose** | **trehalose** | **fructose** | **glucose** | **sucrose** | **data transformation** | **F _[9, 37]_** | ***p* value** |
| sucrose | 2.0 ± 1.4 ^e^ | 10.6 ± 10 ^e^ | 5.8 ± 1.0 ^e^ | 47.3 ± 16.4 ^d^ | 76.3 ± 6.0 ^cd^ | 102.5 ± 27.9 ^bc^ | 152.8 ± 17.6 ^ab^ | 170.6 ± 22.8 ^a^ | 168.5 ± 36.4 ^a^ | 152.5 ± 11.4 ^ab^ | sqrt | 90.02 | <2×10^-16^ |
| glucose | 0 ± 0 ^e^ | 2.2 ± 2.6 ^cd^ | 0.8 ± 0.5 ^de^ | 0 ± 0 ^e^ | 5.5 ± 0.9 ^bc^ | 8.7 ± 5.8 ^b^ | 20.1 ± 4.9 ^a^ | 24.5 ± 9.2 ^a^ | 27.7 ± 6.9 ^a^ | 23.1 ± 4.5 ^a^ | log | 67.93 | <2×10^-16^ |
| fructose | 0 ± 0 ^d^ | 0 ± 0 ^d^ | 0 ± 0 ^d^ | 0 ± 0 ^d^ | 0 ± 0 ^d^ | 3.9 ± 0.2 ^bc^ | 2.6 ± 1.0 ^c^ | 9.2 ± 2.4 ^a^ | 3.8 ± 2.5 ^bc^ | 5.5 ± 1.1 ^b^ | log | 86.43 | <2×10^-16^ |
| raffinose | 0 ± 0 ^b^ | 0 ± 0 ^b^ | 0 ± 0 ^b^ | 0 ± 0 ^b^ | 0 ± 0 ^b^ | 1.5 ± 0.8 ^a^ | 0 ± 0 ^b^ | 0 ± 0 ^b^ | 0 ± 0 ^b^ | 0 ± 0 ^b^ | log | 34.92 | 2×10^-15^ |
| maltose | 0 ± 0 ^b^ | 0 ± 0 ^b^ | 0 ± 0 ^b^ | 60.1 ± 12.7 ^a^ | 0 ± 0 ^b^ | 0 ± 0 ^b^ | 0 ± 0 ^b^ | 0 ± 0 ^b^ | 0 ± 0 ^b^ | 0 ± 0 ^b^ | log | 1548 | <2×10^-16^ |
| ? | 0 ± 0 ^b^ | 0 ± 0 ^b^ | 0 ± 0 ^b^ | 0 ± 0 ^b^ | 0.44 ± 0.04 ^a^ | 0 ± 0 ^b^ | 0 ± 0 ^b^ | 0 ± 0 ^b^ | 0 ± 0 ^b^ | 0 ± 0 ^b^ | no | 495.9 | <2×10^-16^ |
| mellibiose | 0 ± 0 ^b^ | 0 ± 0 ^b^ | 0 ± 0 ^b^ | 0 ± 0 ^b^ | 0 ± 0 ^b^ | 0.6 ± 0.3 ^a^ | 0 ± 0 ^b^ | 0 ± 0 ^b^ | 0 ± 0 ^b^ | 0 ± 0 ^b^ | no | 12.99 | 5×10^-9^ |
| galactose | 0 ± 0 ^b^ | 151.9 ± 65.0 ^a^ | 0 ± 0 ^b^ | 0 ± 0 ^b^ | 0 ± 0 ^b^ | 0.4 ± 0.5 ^b^ | 0 ± 0 ^b^ | 0 ± 0 ^b^ | 0 ± 0 ^b^ | 0 ± 0 ^b^ | no | 15.95 | 3×10^-10^ |
| ? | 0 ± 0 ^b^ | 0 ± 0 ^b^ | 2.4 ± 0.7 ^a^ | 0 ± 0 ^b^ | 0 ± 0 ^b^ | 0 ± 0 ^b^ | 0 ± 0 ^b^ | 0 ± 0 ^b^ | 0 ± 0 ^b^ | 0 ± 0 ^b^ | no | 41.36 | <2×10^-16^ |
| ? | 0 ± 0 ^c^ | 0 ± 0 ^c^ | 2.8 ± 0.4 ^a^ | 0 ± 0 ^c^ | 1.6 ± 0.4 ^ab^ | 2.5 ± 1.9 ^ab^ | 1.4 ± 0.4 ^ab^ | 1.1 ± 0.8 ^b^ | 1.3 ± 0.5 ^ab^ | 1.1 ± 0.3 ^ab^ | log | 12.85 | 6×10^-9^ |
| mannitol | 0 ± 0 ^b^ | 0 ± 0 ^b^ | 234.2 ± 12.0 ^a^ | 0 ± 0 ^b^ | 0 ± 0 ^b^ | 0 ± 0 ^b^ | 0 ± 0 ^b^ | 0 ± 0 ^b^ | 0 ± 0 ^b^ | 0 ± 0 ^b^ | sqrt | 5654 | <2×10^-16^ |
| sorbitol | 0 ± 0 ^b^ | 0 ± 0 ^b^ | 0 ± 0 ^b^ | 0 ± 0 ^b^ | 19.6 ± 1.8 ^a^ | 0 ± 0 ^b^ | 0 ± 0 ^b^ | 0 ± 0 ^b^ | 0 ± 0 ^b^ | 0 ± 0 ^b^ | sqrt | 1754 | <2×10^-16^ |
| total soluble carbohy-drates | 2.0 ± 1.4 ^d^ | 164.6 ± 75.5 ^abc^ | 246.0 ± 13.1 ^a^ | 107.4 ± 28.8 ^c^ | 103.4 ± 5.7 ^c^ | 120.0 ± 30.8 ^bc^ | 176.9 ± 21.6 ^abc^ | 205.4 ± 29.6 ^ab^ | 201.4 ± 44.3 ^ab^ | 182.2 ± 16.9 ^ab^ | sqrt | 35.64 | 1×10^-15^ |
| starch | 407 ± 136 ^abc^ | 729 ± 123 ^a^ | 218 ± 35 ^bc^ | 178 ± 19 ^c^ | 444 ± 121 ^abc^ | 493 ± 188 ^abc^ | 522 ± 133 ^ab^ | 658 ± 120 ^a^ | 582 ± 132 ^a^ | 598 ± 174 ^a^ | no | 7.168 | 6×10^-06^ |

4-months cultivation; 100 mM monosaccharides, 50 mM disaccharides, 33.3 mM raffinose. Means ± SD and ANOVA results are given, superscript letters indicate significantly different groups of data according to the Tukey-Kramer test (α = 0.05).

**Supplementary Table 1.** Effect of soluble carbohydrate type in the medium on the nonstructural carbohydrate content in *D. majalis* protocorms.

| **carbohydrate detected [μg mg^-1^ dry weight]** | **treatment** | | | | **statistics** | | |
| --- | --- | --- | --- | --- | --- | --- | --- |
|  | **glucose** | **sugar free** | **trehalose + validamycin A** | **trehalose** | **data transformation** | **F  [3, 9]** | ***p* value** |
| trehalose | 1.42 ± 0.59 ^c^ | 0 ± 0 ^c^ | 89.5 ± 13.43 ^a^ | 12.73 ± 5.74 ^b^ | sqrt | 151.5 | 5×10^-8^ |
| sucrose | 136.03 ± 16.75 ^a^ | 16.28 ± 7.11 ^b^ | 21.83 ± 1.70 ^b^ | 105.95 ± 20.93 ^a^ | sqrt | 53.35 | 5×10^-6^ |
| glucose | 26.34 ± 8.52 ^a^ | 0 ± 0 ^b^ | 1.17 ± 0.85 ^b^ | 13.76 ± 2.22 ^a^ | sqrt | 48.94 | 7×10^-6^ |
| fructose | 2.92 ± 0.92 ^a^ | 0 ± 0 ^b^ | 0 ± 0 ^b^ | 1.63 ± 0.19 ^a^ | sqrt | 100.8 | 3×10^-7^ |
| ? | 1.24 ± 0.06 ^b^ | 0 ± 0 ^c^ | 5.21 ± 0.16 ^a^ | 2.22 ± 0.64 ^b^ | no | 111.8 | 2×10^-7^ |
| total soluble carbohydrates | 167.94 ± 26.16 ^a^ | 16.28 ± 7.11 ^b^ | 117.70 ± 13.26 ^a^ | 136.28 ± 18.09 ^a^ | no | 37.22 | 2×10^-5^ |

**Supporting Table 2.** Effect of trehalase inhibition by validamycin A on soluble carbohydrate contents in *D. majalis* protocorms after 4-months cultivation.
Means ± SD and ANOVA results are given. Superscript letters indicate significantly different groups of data according to the Tukey-Kramer test (α = 0.05).

| **carbohydrate detected  [g l^-1^]** | **treatment** | | | | **statistics** | | |
| --- | --- | --- | --- | --- | --- | --- | --- |
|  | **glucose** | **sugar free** | **trehalose + validamycin A** | **trehalose** | **data transfor-mation** | **F_[3, 21]_** | ***p* value** |
| trehalose | 0 ± 0 ^c^ | 0 ± 0 ^c^ | 20.61 ± 0.87 ^a^ | 14.92 ± 2.60 ^b^ | no | 177.6 | 4×10^-15^ |
| glucose | 13.69 ± 2.94 ^a^ | 0 ± 0 ^b^ | 0 ± 0 ^b^ | 1.92 ± 0.92 ^b^ | no | 77.29 | 2×10^-11^ |
| total soluble carbohydrates | 13.69 ± 2.94 ^b^ | 0 ± 0 ^c^ | 20.61 ± 0.93 ^a^ | 16.85 ± 1.90 ^ab^ | no | 65.9 | 7×10^-11^ |

**Supporting Table 3.** Effect of trehalase inhibition by validamycin A on the soluble carbohydrate content in medium after 4 months of *D. majalis* protocorm cultivation.
Means ± SD and ANOVA results are given, superscript letters indicate significantly different groups of data according to the Tukey-Kramer test (α = 0.05).

| Plant material | n (separate incubations / sections total) | time of incubation [hours] | % of positively stained sections |
| --- | --- | --- | --- |
| trehalose-supported asymbiotic protocorm | 3/50 | 6-7 | 92^a^ |
| sucrose-supported asymbiotic protocorm | 3/50 | 6-7 | 11.1^b^ |
| glucose-supported asymbiotic protocorm | 3/50 | 6-7 | 6.4^b^ |
| symbiotic protocorms | 4/48 | 0.5 | 95.8 |
| mycorrhizal root parts | 5/50 | 0.5 | 88^a^ |
| non-mycorrhizal root parts | 3/30 | 0.5 | 3.3^b^ |

**Supporting Table 4.** Times of incubation and number of replicates of histochemically stained sections of *D. majalis*.
Incubations were checked regularly and stopped when coloration was visible in positive reactions. Roots from at least three different plants or protocorms from at least three different dishes were used for each reaction. Control incubations without trehalose were completely free of trehalase activity (3 separate reactions, 30 sections total for each variant). Superscript letters indicate significant differences in proportions of positively stained sections according to the generalized linear models (GLM) with binomial distribution.
